# Why more junctions do not yet deliver: interconnection challenges in perovskite multijunction solar cells

**DOI:** 10.1039/d6ee01631f

**Published:** 2026-06-18

**Authors:** Rik Hooijer, Sunwoo Kim, Doyun Im, Cong Chen, Dewei Zhao, Sangwook Lee, Erkan Aydin

**Affiliations:** a Department of Chemistry and Center for NanoScience (CeNS), Ludwig-Maximilians-Universität (LMU) Munich 81377 Germany erkan.aydin@cup.uni-muenchen.de; b School of Materials Science and Engineering, Kyungpook National University (KNU) Daegu 41566 Republic of Korea wook2@knu.ac.kr; c College of Materials Science and Engineering & Engineering Research Center of Alternative Energy Materials & Devices, Ministry of Education, Sichuan University Chengdu China dewei.zhao@scu.edu.cn

## Abstract

Single-junction photovoltaic technologies are approaching their practical efficiency limits. Perovskite-based multijunction solar cells offer a path beyond these limits through reduced thermalization losses and improved spectral utilization. Although tandem architectures are not new to the photovoltaic community, perovskite-based tandems have, for the first time, opened a realistic path toward gigawatt-scale deployment of this technology. Over the past decade, remarkable progress has been achieved, and the technology is now moving steadily toward industrial scaling. Beyond complementing crystalline silicon, perovskites constitute a versatile tandem device component that can be implemented in fully perovskite-based architectures, fabricated on diverse substrates, and even extended to triple or quadruple-junction configurations. However, this advancement introduces new technological challenges, particularly in the interconnection of perovskite sub-cells. Achieving reliable electrical coupling while maintaining interfacial and structural integrity is challenging, as high-efficiency devices often involve multiple solution-processed layers, where similar solvents used in adjacent sub-cells can cause interlayer dissolution or chemical degradation. Furthermore, film imperfections such as cracks, pinholes, and delamination – often driven by mechanical stress and accumulated strain – emerge frequently during fabrication and scale-up and require careful management. This perspective examines these interconnection-related challenges and charts future directions in perovskite-based multijunction solar cells beyond the general electronic view of recombination junctions.

## Abstract 2

Perovskite multijunction solar cells promise high efficiencies, yet progress beyond two-junctions is limited by the recombination junction. We benchmark RJ architectures and propose a framework to guide scalable multijunction development.

## Graphical abstract

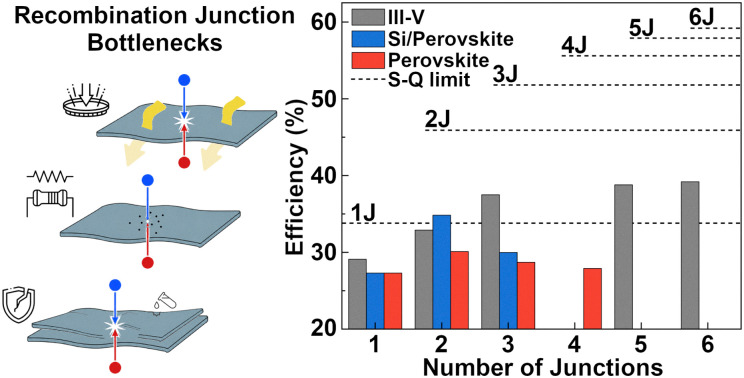

**Broader context**

As traditional single-junction solar cells approach their theoretical efficiency limits, perovskite-based multijunction architectures have emerged as a transformative pathway toward high-efficiency, gigawatt-scale photovoltaic deployment. While the field has seen rapid progress in dual-junction tandems, extending these configurations to triple or quadruple junctions introduces significant mechanical and chemical hurdles. This perspective shifts the focus from purely electronic considerations to the critical physical challenges of sub-cell interconnection, such as interlayer dissolution during solution processing and strain-induced film defects like pinholes and delamination. By addressing these interfacial and structural integrity issues, this work provides a strategic roadmap for the development of robust, next-generation multijunction devices. These insights are essential for transitioning advanced perovskite architectures from laboratory-scale breakthroughs to durable, industrially viable energy solutions.

## Introduction and key questions

Perovskite-based multijunction (tandem) solar cells mark a significant advancement over conventional single-junction (1J) PV technologies such as crystalline silicon, by enabling broader solar spectrum utilization and minimizing thermalization losses.^1^ Importantly, higher power conversion efficiencies (PCEs) can be achieved with potentially minimal added cost due to the convenience of processing perovskite sub-cells, crucial for industrial adoption. They are steadily advancing toward commercialization, with perovskite–silicon tandems currently the closest to market deployment. In parallel, thin-film tandems, and particularly all-perovskite architectures, are following a similar trajectory. Other promising perovskite-based tandem configurations include perovskite–Cu(In,Ga)Se_2_ (CIGS) and perovskite–organic devices.^2,3^

At present, there is no clear consensus on whether monolithic or mechanically stacked tandems will ultimately dominate. However, the research community has placed greater emphasis on monolithic configurations, in which the perovskite sub-cell is directly integrated in series with the bottom sub-cell through an interconnecting recombination junction (RJ). This monolithic integration approach is also the focus of this article, as the efficient electrical and optical coupling of the sub-cells *via* the RJ remains a central technological challenge. As of today, perovskite–silicon tandems have been reported with PCEs close to 35%^4,5^ for 1 cm^2^ active areas with a projected practical limit exceeding 38–39%,^6,7^ while all-perovskite tandems have exceeded 30% PCE for ∼0.05 cm^2^ and 28.5% for 1 cm^2^ active areas.^8^

**Fig. 1:**
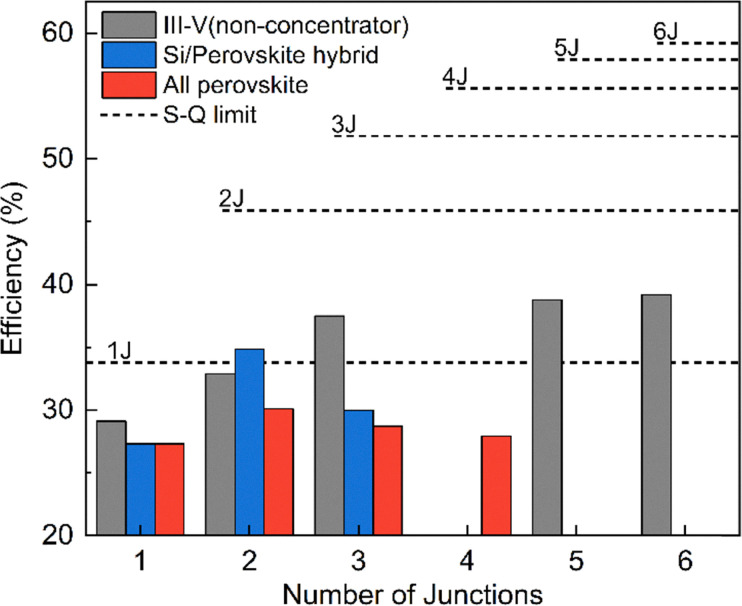
Shockley–Queisser (S–Q) limits for multijunction solar cells concerning the number of RJs. Reported values highlight the increasing gap towards the efficiency limit for a higher number of RJs and the gap to the III–V technologies.^9–12^

Despite the clear efficiency advantages of triple- and quadruple-junction architectures demonstrated in III–V photovoltaics, perovskite-based multijunction cells remain predominantly limited to two junctions (Fig. 1). Although triple-junction cells offer the potential for higher PCEs, this promise has not yet been realized, and the effect becomes more pronounced in larger-area devices, highlighting the challenges of forming efficient RJs in thin-film-based tandem architectures.

Several earlier reviews have addressed RJ design within particular scopes: de Bastiani *et al.* laid the conceptual foundation for monolithic perovskite tandems,^13^ subsequent articles have treated all-perovskite architectures,^14–16^ perovskite/silicon architectures,^17–19^ and most recently RJ progress across multiple multijunction families.^20,21^ Nonetheless, rapid progress during 2024–2026 has revealed RJ bottlenecks that become visible only in record-efficiency devices or under scale-up, requiring RJ-specific, cross-family comparison of reporting metrics that remains largely absent from the literature.

This perspective therefore benchmarks RJ architectures across perovskite/silicon, all-perovskite (2J, 3J, 4J), perovskite/perovskite/silicon, perovskite/CIGS, and perovskite/organic multijunction cells. We argue that the RJ should be treated as a decisive bottleneck rather than as a secondary interlayer, and that architectural simplification is central to reconciling efficiency, scale-up, and reliability. Our conclusions are strongest for p–i–n thin-film RJs, which dominate current high-efficiency demonstrations, and may not fully generalize to n–i–p, mechanically stacked or four-terminal architectures.

## State-of-the-art recombination junctions

In monolithically integrated multijunction solar cells, RJs are key components that opto-electrically couple the sub-cells and govern the overall device efficiency.^13^ An ideal RJ must fulfil several stringent requirements, including appropriate energy band alignment of the electron transport layer (ETL), hole transport layer (HTL), and conductive interlayer (*e.g.*, transparent conductive oxide (TCO)) to enable efficient carrier recombination. The ETL/HTL interface should facilitate rapid electron–hole recombination while minimizing resistance and voltage losses. In addition, the RJ must exhibit high optical transparency to transmit light to the underlying sub-cells, as well as chemical and thermal stability and chemo-mechanical robustness to withstand solution processing of subsequent layers.^22^

State-of-the-art RJs frequently adopt multilayer architectures, which in p–i–n devices typically consist of ETLs from organic materials such as C_60_, PC_60_BM, HTLs such as poly(3,4-ethylenedioxythiophene):poly(styrenesulfonate) (PEDOT:PSS), or inorganic materials such as atomic-layer-deposited (ALD) SnO_2_, and conductive layers including metals (*e.g.*, Au) or TCOs.^23^

At present, the field strongly favours p–i–n configurations.††100 000 results *vs.* 3000 results based on a web of science search with the keywords “perovskite AND tandem OR multijunction AND p–i–n OR inverted” *vs.* “perovskite AND tandem OR multijunction AND n–i–p”. This is due to a combination of factors, such as the low temperature processability, the HTL self-assembled molecule (SAM) compatibility, and better Sn^2+^ stability. Additionally, materials used in n–i–p configurations, such as spiro-OMeTAD or PTAA as HTLs, are not sufficiently transparent, thermally stable, or deposition compatible with the completed bottom sub-cell and often require problematic doping protocols.^18^ Organic alternatives have been demonstrated,^24^ and inorganic alternatives partially address the concerns, but introduce their own interfacial recombination penalties, such as redox-driven interfacial degradation for NiO_*x*_ and morphological challenges for CuSCN.^25,26^ Hence, this perspective is mostly focused on p–i–n configurations.

**Fig. 2:**
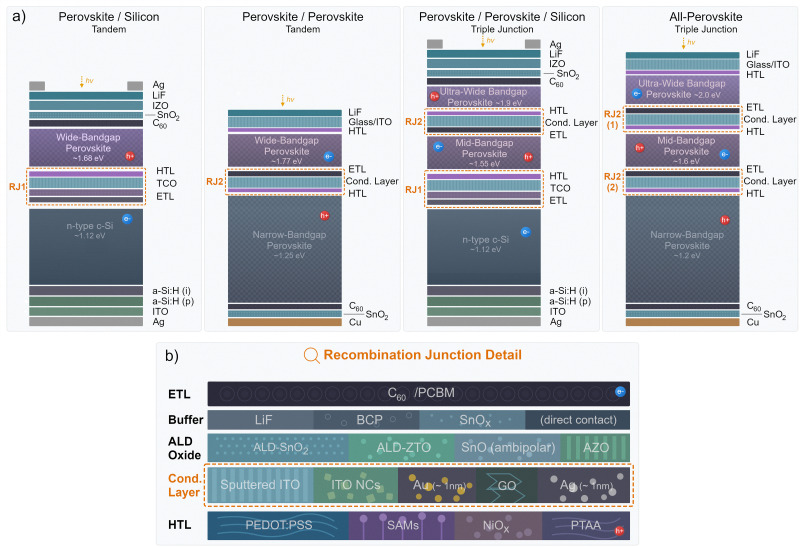
(a) Schematics of the solar cell architectures for perovskite/silicon tandem, perovskite/perovskite tandem, perovskite/perovskite/silicon triple junction, and all-perovskite triple junction solar cells in p–i–n configuration. (b) RJ in a detailed view with the most common material options.

The structural configurations and materials of state-of-the-art RJs are illustrated in Fig. 2. Many materials are multifunctional; for example, SnO_2_ acts both as a buffer layer to prevent sputter-induced damage and/or solvent penetration, and as an electron-selective contact. This multifunctionality is advantageous but also complicates the engineering process. Even with this progress, several persistent failure modes still motivate a closer examination of the limitations of each layer.

## Challenges in current RJ architectures

While significant progress has been made, persistent material and processing bottlenecks in RJ design remain a challenge across the research community, highlighting the need for next-generation architectures. These challenges span all-perovskite and perovskite/silicon tandem devices, with limitations evident in each layer from the n-side to the p-side of the junction.

**Fig. 3:**
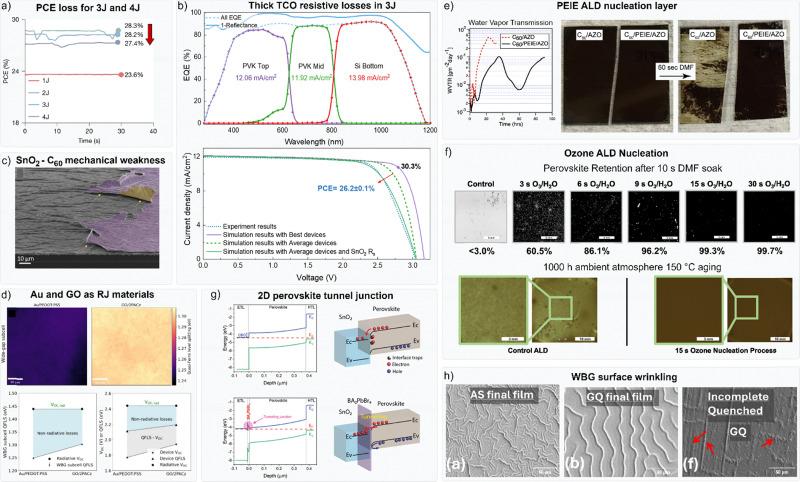
(a) (i) *JV* curves of optimized single-junction, double-junction, triple-junction, and quadruple-junction cells. Reproduced from ref. 10 with permission from Springer Nature,^10^ copyright 2025. (b) EQE of a triple-junction solar cell (top) and the linear technology simulation program with integrated circuit emphasis (LTspice) simulation results based on top-level and average-level single-junction solar cells without and with series resistance (*R*_s_) between top- and mid-perovskite solar cells (bottom). Reproduced from ref. 41 with permission from Elsevier,^41^ copyright 2024. (c) False-coloured tilted SEM image demonstrating the peel-off between C_60_ and SnO_2_. The peeled surface presents the typical wrinkles of the perovskite surface. Reproduced from ref. 40 permission from ACS,^40^ copyright 2022. (d) Quasi Fermi level splitting (QFLS) maps of perovskite dual-junction devices with (i) Au/PEDOT:PSS and (ii) GO/2PACz as the recombination layer with (iii) and (iv) their respective QFLS losses, calculated from individual sub-cell QFLS, respectively. Reproduced from ref. 48 with permission from ACS,^48^ copyright 2025. (e) Water vapor transmission rate of 25 nm AZO as a function of time grown on bare C_60_ and C_60_ functionalized with a PEIE nucleation layer (left). FA_0.6_Cs_0.3_DMA_0.1_PbI_2.4_Br_0.6_ perovskite sample split in two coated with 30 nm C_60_/25 nm AZO on the left and 30 nm C_60_/PEIE/25 nm AZO on the right (middle). The same perovskite sample after 60 s of dimethylformamide (DMF) exposure (right). Reproduced from ref. 22 with permission from Elsevier,^22^ copyright 2019. (f) Perovskite film retention after a 10 s DMF exposure for glass/perovskite/C_60_/ALD SnO_*x*_ with increasing duration of ozone exposure applied after the initial 40 cycles of SnO_*x*_ capping layer growth (top). Glass/perovskite/C_60_/ALD SnO_*x*_ films after aging for 1000 h at 150 °C in an ambient oven using a control SnO_*x*_ and ozone-nucleated (15 s) SnO_*x*_ ALD process (bottom). Reproduced from ref. 32 with permission from Elsevier,^32^ copyright 2023. (g) Band diagram schematic of a control device and a device employing a 2D perovskite layer as a tunnelling junction. Reproduced from ref. 49 with permission from Wiley-VCH,^49^ copyright 2024. (h) WBG perovskite surface wrinkling employing an antisolvent technique or a gas quenching technique. Reproduced from ref. 50 with permission from ACS,^50^ copyright 2025.

All-perovskite tandem solar cells typically employ p–i–n configurations with RJ stacks such as SnO_2_/Au or ITO/PEDOT:PSS.^23,27–29^ For perovskite/silicon dual-junction tandems, RJ design typically adopts established structures such as n-Si (amorphous or nanocrystalline)/TCO/HTL (*e.g.*, SAM, NiO_*x*_, or modified NiO_*x*_), where interface quality is crucial for high-performance devices.^11^ Perovskite/perovskite/silicon triple-junction tandems incorporate two recombination junctions: RJ1 (Si–PK RJ), connecting the silicon bottom sub-cell to the middle-bandgap perovskite sub-cell, and RJ2 (PK–PK RJ), linking the middle-bandgap and top-bandgap perovskite sub-cells. While RJ1 design follows the same rationale as perovskite/silicon dual-junction tandems, RJ2 is more challenging due to its thin-film/thin-film integration between two perovskite sub-cells. If robust RJ2 designs can be established, higher-junction integration (*e.g.*, 4J concepts) becomes more feasible; however, current 4J demonstrations remain largely proof-of-concept, even showing efficiency losses compared to 2J and 3J devices (Fig. 3a).

In RJ2s, dense ALD–SnO_2_ buffer layers are widely introduced on top of C_60_ to suppress solvent penetration and mitigate sputter-induced damage to the underlying perovskite layers during subsequent processing steps. This strategy reflects the excellent electron-selective properties of C_60_ and SnO_2_, combined with the superior barrier functionality of ALD–SnO_2_, which shows promise with tuneable oxygen vacancies enabling better charge extraction and environmental stability.^30,31^ However, the deposition of high-quality SnO_2_ films on C_60_ remains intrinsically challenging, as C_60_ surfaces are highly hydrophobic and chemically inert, resulting in poor nucleation during the initial ALD cycles and non-uniform film growth.^32–34^ To compensate for this limitation, comparatively thick SnO_2_ layers of 10–20 nm are often employed, which in turn introduce additional parasitic absorption interfering with current matching between sub-cells,^35,36^ increased contact resistivity, conduction band offsets that need tuning,^37^ and an inherently weak mechanical interface (Fig. 3b and c).^38–40^ Importantly, the optical penalty of thicker RJ barrier layers becomes increasingly severe as the number of junctions increases because additional parasitic absorption directly reduces the current of the current-limiting sub-cell. As a simple power-density estimate, an RJ-related current loss of 0.1 mA cm^−2^ translates to 0.15–0.2 percentage points of PCE loss in a 2J cell at 1.5–2.0 V maximum power, with the penalty expected to scale further in 3J+ devices due to their higher operating voltages and stricter current-matching constraints.^41,42^

Beyond nucleation challenges, C_60_ can induce voltage losses and increased contact resistance due to limitations associated with surface charge heterogeneity and weak van der Waals bonding with the underlying perovskite layers.^43^ These effects become more pronounced when the underlying perovskite film is chemically and electronically non-uniform: heterogeneous charge distributions broaden the energy levels of charge-transfer states, leading to low *V*_oc_,^44–46^ while weak interfacial bonding results in non-uniform electrical contact and consequently higher interfacial resistance.^47^ Moreover, the relatively weak interfacial adhesion between C_60_ and adjacent layers compromises mechanical robustness and increases susceptibility to delamination under thermal and processing-induced stress (Fig. 3c).^40^

On top of the SnO_2_ buffer layer, TCOs (∼40–70 nm) are commonly employed to provide sufficient conductivity for electron–hole recombination and to function as an auxiliary solvent barrier. However, the thick TCOs introduce optical penalties, morphological cracking, shunting risks, sputter damage, and reduced mechanical adhesion.^40,51^ These issues become increasingly severe during scale-up, as pinhole formation in wide-bandgap (WBG) perovskite films amplifies leakage currents and compromises device yield.^52^ As an alternative to thick TCO layers, ultrathin Au nano-islands (∼1 nm) have been widely adopted (Fig. 3d); however, this approach introduces different challenges, including optical losses by reflection and degradation paths *via* Au diffusion into adjacent layers, causing irreversible trap states and shunt paths.^48,53–55^

On top of the conducting layer, *i.e.*, the p-type side of the RJ, the most employed HTL is PEDOT:PSS, due to its favourable compatibility with narrow-bandgap (NBG) perovskites, *i.e.*, a well-matched valence band maximum, its solution processability, and protective thickness. However, PEDOT:PSS is both acidic and hygroscopic, accelerating Sn oxidation in NBG perovskites and severely degrading device stability.^27,56,57^ In addition, surface inhomogeneity within PEDOT:PSS reduces electron-blocking capability, while polar solvents used in perovskite precursor solutions erode the layer, weakening hole extraction at the HTL/perovskite interface.^58^ It also features a non-negligible parasitic absorption.^59^ To address these issues, alternative HTLs such as self-assembled monolayers (SAMs), BCF-doped PEDOT,^60^ poly[bis(4-phenyl)(2,4,6-trimethylphenyl)amine] (PTAA), poly(3-hexylthiophene) (P3HT), and metal oxides, including NiO_*x*_, SnOCl, and SnO_*x*_, have been explored. Nevertheless, perovskites often suffer from poor wettability on organic HTL surfaces,^61,62^ whereas oxide-based HTLs can undergo detrimental redox reactions and exhibit process-dependent instability.^63,64^ Recently, NiO_*x*_ layers or NiO_*x*_/SAM combinations function as hole-selective contacts. For NiO_*x*_ in particular, nanoparticle-based films demonstrate improved tolerance to mechanical and thermal stress,^11^ while sputtered amorphous NiO_*x*_ yields denser, pinhole-free layers that more effectively block solvent infiltration, underscoring the strong dependence of HTL performance on deposition methodology.

## Emerging materials and processing strategies

Recent RJ strategies increasingly target several constraints at once: charge extraction and recombination, solvent and sputter protection, mechanical stability, and scalable deposition. We therefore group them into three tiers: approaches already used in record-class devices with scalable deposition routes; functional approaches with unresolved process or deposition trade-offs; and concepts that are promising but require validation in large-area or higher-junction devices.

Tier one strategies that underpin record-class perovskite multijunction cells at areas of 1 cm^2^ and above using scalable methods are sputtered TCOs and ALD SnO_2_. TCO-based interlayers remain central to many high-performing monolithic architectures because they provide lateral conductivity, solvent protection, SAM bonding sites and enable recombination. Although sputter-induced damage to the underlying perovskite remains a known issue, it is manageable at scale and sputtering is broadly established. ALD SnO_2_ has likewise become an essential buffer/barrier layer, with the nucleation challenge on hydrophobic C_60_ addressed through functionalization with PEIE or carboxyl-terminated molecules,^22,31,65^ or *in situ* ozone functionalization (Fig. 3e and f), improving its coverage and density.^32^

Tier two strategies are functional but face unresolved deposition or process trade-offs. SAM-based contacts are widely used in perovskite–silicon tandems, though less common in thin-film tandems such as all-perovskite devices. When deposited on TCO layers, SAMs form covalent or ionic bonds, producing highly stable and densely packed interfacial layers that enhance device performance.^66,67^ Phosphonic acid groups provide strong bi-/tridentate anchoring, while silane-based SAMs can enable long-term stability *via* Si–O–Si crosslinking, albeit with enhanced risk of hydrolysis.^68,69^ However, spin-coating remains poorly suited for high-throughput manufacturing, and scalable vapor-phase,^70^ slot-die,^62^ and roll-coating routes for SAMs are emerging but not standardized.^71^ Reproducibility, surface hydroxylation control and defect-free coverage at scale remain principal challenges.^72^ Direct perovskite surface functionalization could also offer improved direct SAM bonding, bypassing the TCO–SAM step.

Alongside SAMs, several refinements within the sputter/TCO framework sit in this tier. Soft-sputtering protocols employing radio-frequency sources, decreased powers, and increased pressures lower particle energy and minimize ion-induced damage,^73,74^ and reactive plasma deposition was shown as a viable alternative method for damage-free RJs.^75,76^ Both approaches are yet to be demonstrated on all-perovskite cells. Solution-processed metal-oxide nanoparticle dispersions provide a scalable approach to protecting against sputter damage, although their application is limited to smooth (planar) front surfaces.^77^ Indium-free approaches have also drawn interest from a supply- and cost-considering point of view. Zinc tin oxide was demonstrated successfully,^78,79^ while other earth-abundant TCOs, such as aluminium-doped zinc oxide or doped titanium oxide, might be worthwhile to explore further.^80–83^ Graphene oxide (GO) with an average ∼93% transmittance across 400–1100 nm has been proposed as an RJ material in all-perovskite cells (Fig. 3d). GO nanosheets, rich in hydroxyl and carboxyl groups, offer functional binding to SAMs or metal oxides, with high conductivity when tuned appropriately.^48^ Deposited from dispersions, they can effectively cover pinholes in large area WBG perovskite films. However, the transparency-conductivity trade-off, interfacial stress management, and scalability require further optimization.^84,85^

Tier three strategies are functionally promising but not yet validated at larger area, high efficiency or different multijunction families and 3J+ cells. Fully molecular/organic RJs, demonstrated with evaporated n-doped C_60_ and p-doped arylamine, offer a way without TCO entirely. This is a valuable approach for textured or rough surfaces, mitigating shunting effectively through a decreased lateral conductivity of the organic layers.^86^ Another way to bridge the perovskite surface directly is low-dimensional perovskites (LDPs). They offer improved chemical stability, bandgap tunability, and interfacial passivation, and importantly, they can form ionic bonds between the perovskite and transport layers. Electronically, they can act as tunnelling layers (Fig. 3g), while also suppressing halide segregation in WBG compositions.^49,87–89^ Their application as phase-pure 2D, mixed halide, or Sn–Pb compositions specifically engineered as ultrathin RJ interface materials remains underexplored. Overall, reducing the number of layers in RJ stacks can effectively lower process complexity and improve stability while removing PEDOT:PSS. The multifunctionality of ALD SnO_2_ and ITO nanocrystals as stable and conductive buffer layers for example contributes to RJ simplification.^90,91^ However, neither approach has been validated for reproducibility across multiple fabrication sites or at areas exceeding 1 cm^2^.

Table 1 highlights representative RJ architectures selected for RJ-specific innovation rather than historical priority or champion efficiency alone, with a detailed cross-family benchmark provided in Table S1. The selected entries show that recent perovskite/silicon progress largely converged towards and refines the mature TCO/SAM backbone through targeted solutions to SAM layers, sputter damage, damp-heat instability, textured-surface coverage, and non-TCO contact design. In contrast, all-perovskite and 3J architectures have not converged towards one architecture and still rely heavily on more complex C_60_/SnO_*x*_/conductive-layer/HTL recombination stacks, where RJ2 design, repeated thin-film interconnection, optical loss, and current matching become increasingly limiting as junction count rises. Simplified and innovative RJ concepts remain mostly demonstrated at small area or limited stability conditions. Thus, the most informative benchmarks are not simply the highest-efficiency devices, but those that reveal whether an RJ strategy can be validated for area, reproducibility, electrical and optical loss, and operational stress.

**Table 1:** RJ architecture benchmarks in perovskite multijunction solar cells. Entries are selected for RJ-specific innovation and distinction, rather than champion performance alone. A full overview table is given as Table S1. Tiers indicate the maturity of the full RJ strategy highlighted in each row, while the stack column reports the full RJ-relevant layer sequence

Tier	Family	RJ stack	RJ strategy	PCE/area	Comment	Source
1	PK/Si 2J	a-Si/nc-SiO_*x*_/ITO/CuSCN/PK	Inorganic CuSCN grains replace SAM	31.02% stab./0.997 cm^2^	4 cm^2^ demonstrated, blade-coatable	Kan 2024^92^
2		a-Si/nc-Si(n)/nc-Si(p)/spiro-TTP/PK	nc-Si tunneling junction; non-TCO RJ route for PK/Si	29.4% stab./0.500 cm^2^	Lower PCE, small area, spin-coated	Liu 2024^93^
2		a-Si/nc-Si/IZO/CL-SAM/PK	Cross-linked SAM directly addresses SAM thermal disorder	33.61% stab./1.00 cm^2^	SAM spin-coated	Zhang 2025^94^
2		a-Si/nc-SiO_*x*_/RPD ICO:H/Me-4PACz/PK	Reactive-plasma-deposited TCO; cerium and hydrogen doped	33.2% stab./1.00 cm^2^	SAM spin-coated	Wang 2025^75^
2		a-Si/nc-SiO_*x*_/ITO/ns-SiO_*x*_/2PACz/PK	Localized submicron nanosphere contacts on industrial texture	33.08% stab./1.00 cm^2^	Mostly spin-coated	Zhang 2025^95^
3		a-Si/n-C_60_/p-TaTm (p)/TaTm-SC9/TaTm/PK	Fully organic, evaporated	22.19%/n.r.	Low PCE, no stability or large area validation	Chozas-Barrientos 2025^86^
3	All-PK 2J	C_60_/SnO_2−*x*_/PK	Simplified two-layer RJ; no Au, no TCO	24.6%/0.059 cm^2^	Small area, early stage, low PCE	Yu 2020^91^
2		C_60_/SnO_*x*_/Au/PEDOT:PSS/SA/PK	Standard SnO_*x*_/Au/PEDOT:PSS layers	30.1% stab./0.049 cm^2^	Small area, spin-coated, with Au/PEDOT:PSS	Lin 2025^9^
2		C_60_/GO/2PACz/PK	GO replaces Au + PEDOT:PSS; QFLS-resolved RJ losses	23.3%/0.12 cm^2^	Low PCE, spin-coated, small area	Fitzsimmons 2025^48^
2		C_60_/Cr/ITO/PEDOT:PSS/PK	Thin Cr barrier; ALD-free and Au-free	26.56%/0.09 cm^2^	Low PCE, small area, spin-coated, with PEDOT:PSS	Wei 2025^96^
2		C_60_/SnO_2_/Au/H-bond SAM/PK	SAM with hydrogen-bond network replaces PEDOT:PSS	28.4% stab./0.0686 cm^2^	Small area, spin-coated, with Au	Wang 2026^97^
2	PK/PK/Si 3J	RJ2: C_60_/SnO_*x*_/ZTO/2PACz/PK	Indium-free sputtered ZTO in RJ2	21.9%/1.00 cm^2^	Low PCE, no stability, spin-coated	Heydarian 2025^78^
2		RJ1: nc-Si/ITO/SAM/np-SiO_*x*_/PK	SiO_*x*_ nanoparticles improve current balance in 3J stack	30.02% stab./0.974 cm^2^	Complex dual RJ stack, spin-coated	Artuk 2026^98^
		RJ2: C_60_/SnO_*x*_/IZO/SAM/np-SiO_*x*_/PK				
2	All-PK 3J/4J	All RJs: C_60_/ALD–SnO_*x*_/IZO; PEDOT:PSS for Sn–Pb	Universal repeated thin-film RJ enabling all-PK 3J and 4J demonstrations	3J: 27.28%/1.003 cm^2^; 4J: 27.4%/0.25 cm^2^	Repeated RJs, proof-of-concept, low PCEs	Hu 2025^10^

## Processing and scale-up challenges

In all-perovskite cells, compact SnO_2_ buffer layers of roughly 10–20 nm, processed at ≤100 °C, are often used to prevent solvent infiltration during subsequent deposition of the upper sub-cells.^99^ While effective in protecting the underlying perovskite, these layers introduce contact resistivity and stress accumulation and should be improved to be thinner and more compact. Process conditions, including ALD deposition parameters such as precursor quality, pulse-purge time, and substrate temperature, as well as TCO sputtering parameters such as particle energy, and precursor-related parameters such as additives, solvent types, and annealing protocols, significantly affect the reliability of such barriers. While variations such as spatial ALD can offer increased speed and throughput, it also shifts the regime towards chemical vapor deposition, where, at high speeds, the conformal coating is not as uniform regarding its thickness compared to standard ALD.^100,101^

In all-perovskite multijunction cells, highly concentrated perovskite precursor solutions used for mid- and narrow-bandgap layers frequently induce surface wrinkling on the µm scale, disrupting coating uniformity of solution-based processed thin films, and inducing localized strained areas on subsequent layers, as well as forming resistive domains due to the thickness inhomogeneity.^102^ Gas-quenching and vacuum flashing techniques have been shown to mitigate wrinkling and improve crystal orientation, but further improvements are necessary since the thin RJ layers cannot smooth this out and potentially risk non-uniform thicknesses (Fig. 3h).^50,103–105^

Increasingly multi-layered RJ stacks improve processing compatibility and damage mitigation, but also introduce optical, electrical, and manufacturing trade-offs.^13^ These effects collectively amplify process sensitivity and variability, posing significant barriers to reproducibility and manufacturing scalability. Earlier 3J+ demonstrations showed pronounced area-scaling losses, whereas recent work has narrowed the gap between sub-cm^2^ and 1 cm^2^ devices. This suggests that junction count alone is not the limiting factor; rather film uniformity, pinhole control and RJ continuity govern scale-up.^10^

Long-term RJ stability remains insufficiently characterized, and systematic studies of *T*_80_ lifetimes under damp-heat or thermal cycling conditions are scarce for RJ-specific degradation modes, including halide and metal-ion migration across the RJ, C_60_/SnO_2_ thermomechanical delamination, SAM thermal disordering, and current-mismatch-amplified field screening in the voltage-limited sub-cell.^106,107^ This hinders reliable lifetime predictions for module-level deployment.

Lastly, the emergence of flexible and light-weight substrates and cell types adds a design constraint on the flexibility of the RJs, where their mechanical resilience must also be considered.^75^ Especially, the brittle nature of metal oxides that have low Poisson ratios and large elastic moduli makes them failure points and risky crack initiation sites.^108,109^

## Industrial requirements and pathways to commercialization

For perovskite multijunction cells to transition from laboratory-scale demonstrations to commercial deployment, RJs must be designed with scalability, manufacturability, and long-term reliability in mind. Current approaches demonstrate feasibility but remain insufficiently robust for industrial implementation.^15,91,110–112^ In addition, manufacturing yield is a critical industrial requirement for RJs. Even low densities of pinholes, microcracks, or local delamination can translate into severe shunting and yield loss when scaled to large-area modules. Therefore, industrially viable RJ designs must exhibit a high defect tolerance and process robustness.

Moisture- and ion-induced degradation remains one of the key barriers to module-level reliability. Accordingly, RJ interfaces should rely primarily on intrinsically stable interfacial chemistries and mechanically robust layer stacks, with encapsulation serving as complementary rather than primary protection. The exposure of NBG perovskite as the final absorber layer in all-perovskite cells compounds this challenge, but may be mitigated by fabricating in a switched substrate configuration.^113^ Furthermore, degradation problems after scribing on the module level are another scaling challenge, which needs to be addressed through, for example, ALD–SnO_2_ protective layering.^111^

Other materials, such as gold or complex multilayer stacks (>3 layers), increase cost and further complicate processing. Industrial translation might also require low-cost options, such as carbon-based contacts (*i.e.*, carbon allotropes and derivatives) or conductive polymers (*i.e.*, PEDOT:PSS, PTAA, P3HT, *etc.*), provided their challenges are resolved, and stability is maintained.^114–116^

A further operational stressor with direct implications for the RJ is reverse bias. In perovskite/silicon tandems, the silicon sub-cell's high breakdown voltage shields the perovskite sub-cell,^117^ but only when silicon is current-limiting. Under red-rich spectra or high-albedo spectra, the perovskite becomes current-limiting and reverse-biased.^118^ All-perovskite architectures lack such a shield entirely. Recent multi-scale simulations show that mobile-ion and active-area heterogeneities can localize reverse-bias current into hotspots near pinholes, cracks and RJ inhomogeneities, potentially accelerating metal diffusion (Au), trap formation, and delamination pathways (C_60_/SnO_*x*_).^119^ Cell-integrated bypass concepts offer a complementary device-level route, though their extension to tandems with multiple RJs remains to be demonstrated.^120^

At present, simplified RJ stacks based on ALD–SnO_2_, robust TCOs, and chemically stable SAM layers appear most promising candidates for tandem modules, as these materials currently offer the best balance between optical transparency, process compatibility, scalability, and operational stability. The broader benchmark compiled in Table S1 reinforces this conclusion. Across 79 representative multijunction reports, the highest efficiencies are often not associated with RJ-specific innovation. Many record devices rely on established TCO/SAM or C_60_/SnO_*x*_/conductive-layer/HTL backbones, while performance is improved through absorber development. Genuinely new RJ concepts remain less mature in area, stability or performance. A systematic reporting gap is also revealed: RJ-specific parameters are rarely isolated. The field therefore needs to report RJ-resolved metrics that can be compared across architectures and translated to module qualification targets.

Because RJ performance is rarely reported as a standalone metric, only approximate target ranges can currently be proposed. As a practical benchmark, the contact resistivity should not exceed 1 Ω cm^2^ per RJ. For comparison, III–V tunnel junctions achieve 10^−4^ Ω cm^2^.^121^ Perovskite RJ values remain orders of magnitude higher.^91^ Optical losses from each RJ should be kept below 0.5 mA cm^−2^ current loss, consistent with 15–20 nm C_60_ or PCBM/SnO_2_/ZTO stack loss.^122^ RJ ITO below 20 nm can also recover >1 mA cm^−2^ through reflection optimization.^123^ Mechanical integrity should be reported alongside optical and electrical losses, since weak multilayer interfaces fail during thermal cycling, scribing, lamination and module integration. For C_60_/SnO_2_ stacks, the interface fracture energy has been measured at only 1.2 J m^−2^,^124^ a weak point under thermal-cycling and outdoor-relevant stress, with similar concerns for perovskite/SAM interfaces.^107,124,125^ Targeted interface engineering can raise this to 160 J m^−2^, demonstrating deliberate mechanical robustness is achievable.^124^ These values represent approximate benchmarks until RJ-specific protocols are standardized.^126^

## Potential development areas

Research must advance beyond single-function ETLs and HTLs toward multifunctional materials that combine charge-selective transport, passivation of defects, and protection of underlying layers while maintaining optical transparency, energetic alignment, high stability, processing compatibility with adjacent layers, as well as functionality at both interfaces.^128–131^ The emerging materials discussed above must now be subjected to systematic screening under operational conditions, focused on potential RJ-specific failure modes, for example, fracture energies or adhesion of RJ materials, halide diffusion into the RJ after illumination and temperature cycling, the redox stability under bias and illumination, or current-mismatch tolerance under varying spectral mismatch. The key will be standardized protocols enabling meaningful comparison across architectures with explicit RJ structures.

Reducing RJ stacks to 2–3 layers while preserving device performance is essential for scalable multijunction architectures. Strategies include combining buffer and passivation layers, which protect from subsequent layer processes, such as sputtering or PEDOT:PSS solution, while simultaneously passivating the surface of the underlying layer. Leveraging epitaxial growth to create atomically coherent interfaces between LDPs and perovskites could be an option to achieve that.^127,132,133^ Another option could be removing C_60_ entirely and relying on just SnO_2_, as demonstrated in single junction cells.^30^ Building on the nucleation-promoting interlayers discussed above, an alternative route is oxidized/functionalized C_60_ for high-quality direct ALD TCO growth.^32^

Direct LDP heterojunctions have not yet been demonstrated in perovskite multijunction RJs, either at the 3D absorber/RJ interface or as intra-RJ LDP–LDP stacks. Exploring these alongside other unconventional architectures, such as LDP–SAM, TCO–SAM, or SAM–SAM heterojunctions, using orthogonal processing strategies such as gas-phase deposition or solubility-mismatched solution routes, could open pathways toward more integrated RJ architectures with fewer interfacial constraints. We group them among tier 3 strategies, although they remain long-term visions and proof-of-concept is still required.

For 3J+ devices, reducing cumulative RJ complexity will become increasingly important. Near-term routes include soft-sputtering protocols and co-optimization of optical management with RJ design, particularly where current-matching constraints are severe.^74,134,135^ Longer-term directions include ultrathin or fully organic RJs, direct TCO/perovskite contacts, and SAM-based heterointerfaces enabled by orthogonal processing. Alternative processing routes, including substrate-configured architectures,^113^ may also reduce repeated exposure of fragile perovskite compositions and buried RJs to solvent, thermal, or plasma-induced stress.

**Fig. 4:**
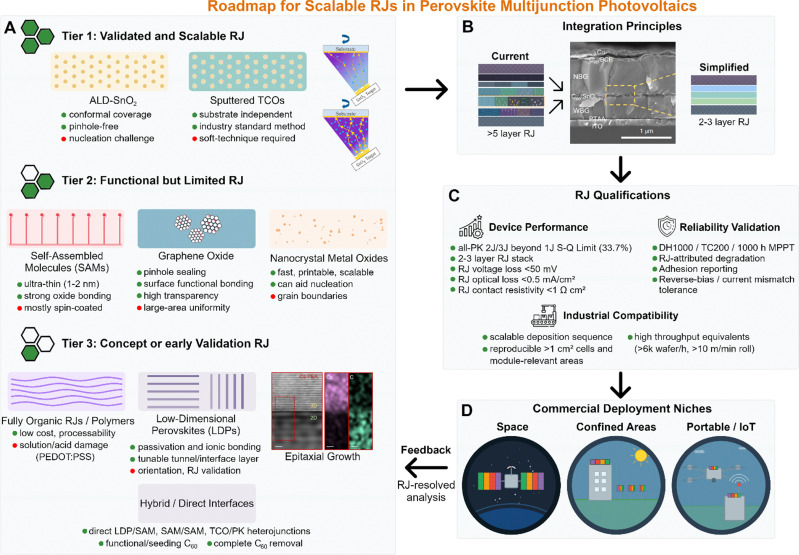
Roadmap for scalable RJs in perovskite multijunction photovoltaics. (A) Emerging RJ materials and processing strategies are grouped by current validation level: tier 1, validated and scalable approaches already used in record-class or module-relevant devices; tier 2, functional approaches with unresolved process reproducibility, or scale-up limitations; and tier 3, concepts requiring broader validation in large-area, high-efficiency, cross-family, or 3J+ devices, or remain long-term and proof-of-concept directions. (B) Candidate strategies should converge toward simplified 2–3-layer multifunctional RJs. (C) These designs must pass RJ-specific qualifications for device performance, reliability validation, and industrial compatibility. (D) Early commercial deployment is expected first in applications where high power per area or power per weight is critical. RJ-resolved analysis and modelling provide feedback for RJ design. The epitaxial growth figure is reproduced from ref. 127 with permission from AAAS,^127^ copyright 2025. The soft sputter figure is reproduced from ref. 74 with permission from Elsevier,^74^ copyright 2026. The C_60_/SnO_2_ image is reproduced from ref. 91 with permission from Springer Nature,^91^ copyright 2020.

III–V multijunction photovoltaics offer benchmark design principles in tunnel-junction design, stress management, junction isolation and interface-resolved reliability, while organic photovoltaics provide complementary lessons in interfacial engineering.^136^ Although direct epitaxy and graded doping are only partially transferable to perovskites,^137^ useful concepts include minimizing RJ widths through controlled layer growth and adapting accelerated aging protocols, such as TC200, DH1000 and 1000 h MPPT, for RJ-specific qualification.^138–141^ The discussed strategies, milestones, and commercialization targets are summarized in Fig. 4.

Advancing RJ designs fundamentally involves dedicated characterization techniques for its specific contribution to the full solar cell. Suns-*V*_oc_ measurements with selective sub-cell illumination can identify shunting pathways through the RJ.^86^ Hyperspectral photoluminescence imaging maps quasi-Fermi level splitting with spatial resolution, revealing losses at the RJ interface.^142^ Cross-sectional transmission electron microscopy combined with energy-dispersive X-ray spectroscopy provides direct visualization of the RJ layer at the nanometer scale.^143^ Emerging techniques, such as transient surface photovoltage (tr-SPV) spectroscopy, can probe charge carrier dynamics at the RJ interfaces,^144^ while spectroscopic ellipsometry can be used to quantify RJ layer-specific parasitic absorption.^145^ Solid characterization protocols will be essential for meaningful comparison between the diverse landscape of RJ architectures, and simulations such as transfer-matrix optical modelling must be included to optimize RJs fully.^42,146^

## Summary

RJs are the decisive bottleneck in the advancement of perovskite multijunction solar cells. They not only connect sub-cells electrically and optically but also dictate manufacturing viability and long-term reliability of multijunction architectures. Current multilayer architectures, from TCO-based stacks to ultrathin metals, GO interlayers, and SAM contacts, have enabled record efficiencies, yet fall short under the compounding demands of scale-up, parasitic absorption, and operational stability.

This perspective has highlighted that the challenge is not merely additive, but each additional junction multiplies the number of interfaces, tightens processing windows, and compounds failure modes in ways that cannot be addressed by optimizing individual layers in isolation. The field's current trajectory of adding layers to protect previous ones is pragmatic but ultimately unsustainable as junction counts increase. The path forward demands multifunctional layers and reduced stack complexity, with materials being designed for the full stack rather than being retrofitted into it, and RJ designs validated not just for peak efficiency but for yield, reproducibility, and lifetime.

We identify three critical milestones to signal genuine readiness for commercialization: (i) demonstrating reliable large-area multijunction cells (>1 cm^2^), (ii) validating long-term reliability under operational stress and RJ-specific accelerated aging protocols that decouple RJ-degradation from absorber-degradation, and (iii) ensuring industrial compatibility with scalable, cost-effective processes and materials. Cross-learning from III–V and organic photovoltaics can inform this effort, but only where parallels are genuine or transferable.

In conclusion, we anticipate that more junctions will eventually deliver, but only if the community treats interconnection engineering with the same intensity and rigor currently applied to absorber optimization. The RJ is the structural, chemical, and electronic backbone of every multijunction device. Its design will ultimately determine whether perovskite multijunction cells remain laboratory achievements or become a manufacturing reality.

## Author contributions

R. H.: writing – original draft, review & editing, conceptualization, visualization, investigation. S. K.: writing – review & editing, visualization, investigation. D. I.: investigation. C. C.: writing – review & editing. D. Z.: writing – review & editing. S. L.: writing – original draft, review & editing, supervision, funding acquisition. E. A.: writing original draft, review & editing, supervision, and funding acquisition.

## Conflicts of interest

There are no conflicts to declare.

## Supplementary Material

EE-019-D6EE01631F-s001

## Data Availability

Supplementary information: it includes Table S1, which benchmarks recombination junction architectures, stacks, and device parameters in perovskite-based multijunction solar cells after the year 2020. See DOI: https://doi.org/10.1039/d6ee01631f. Data sharing is not applicable to this article as no new primary datasets were created or analyzed during the current study.
