# Supplementary material for: Why more junctions do not yet deliver: interconnection challenges in perovskite multijunction solar cells
Source: Energy Environ Sci. 2026 Jun 18;19(14):4582–96. doi: 10.1039/d6ee01631f (PMC13306720; doi:10.1039/d6ee01631f)
Supplement: EE-019-D6EE01631F-s001 [file EE-019-D6EE01631F-s001.pdf]

Supplementary Information (SI) for Energy & Environmental Science.
This journal is © The Royal Society of Chemistry 2026

Benchmark of Recombination Junction Architectures in Perovskite Multijunction Solar Cells

#	1st author / Year	RJ stack (and adjacent layers, manufacture bottom to top)	RJ Deposition	RJ Key innovation	PCE (%)	V_{oc} (V)	FF (%)	J_{sc} (mA/cm²)	Active / Aperture area (cm²)	Cert. body	Stability metrics	DOI	Citation
					(lab if no certified report)								
			Silicon							Perovskite			
1	Mailoa 2015	a-Si / Si / TiO_2 / mp-TiO_2 / PK	PECVD / PECVD / ALD / spin-coat / spin-coat	First Si/PK tandem with tunnel junction	13.7	1.58	75	11.5	1	n.r.	n.r.	10.1063/1.4914179	Mailoa J. et al., Appl. Phys. Lett. 106, 121105 (2015)
2	Bush 2017	a-Si / ITO / NiO_x / PK	PECVD / sputter / spin-coat	not at RJ; ALD/pulsed-CVD SnO_2/ZTO	23.6	1.65	79.0	18.1	1.1 / 1	NREL	only PK SJ reported	10.1038/nenergy.2017.9	Bush K.A. et al., Nat. Energy 2, 17009 (2017)
3	Hou 2020	a-Si / InO_x / NiO_x / PK	PECVD / sputter / sputter / spin-coat	not at RJ: 1-butanethiol limits WBG phase separation	(25.7 stab.)	1.7932	73.66	15.88	0.832	Fraunhofer ISE	100% after 400 h, 40 °C; 100% after 400 h, 85 °C, both MPPT	10.1126/science.aaz3691	Hou. Y et al., Science 367, 1135-1140 (2020)
4	Al-Ashouri 2020	a-Si / nc-SiOx (n) / ITO / Me-4PACz / PK	PECVD / PECVD / DC sputter / spin-coat	SAM on ITO	29.14	1.91	78.87	19.36	1.13 / 1.04	Fraunhofer ISE	>95% after 300 h (MPPT, air, 25 °C, 30-40%RH)	10.1126/science.abd4016	Al-Ashouri A. et al., Science 370, 1300–1309 (2020)
5	Mao 2022	a-Si / ITO / NiO_x 2PACz / PK	RF-PECVD / DC sputter / RF sputter / spin-coat	ITO / NiO_x / SAM enables fully textured Si wafers	28.84	1.794	79.95	20.11	1.2 / 1.2	NIMTT	n.r.	10.1002/adma.202206193	Mao L. et al., Adv. Mater. 34, 2206193 (2022)
6	Mariotti 2023	a-Si / nc-SiOx / ITO / Me-4PACz (+HDPA) / PK	PECVD / PECVD / sputter / spin-coat / spin-coat	not at RJ: PI at PK / C_{60} interface	32.5	1.98	81.18	20.24	~1	JRC-ESTI	80% after 347 h (MPPT, air, 23-32 °C)	10.1126/science.adf5872	Mariotti S. et al., Science 381, 63–69 (2023)
7	Chin 2023	a-Si / nc-SiOx (n,p) / ITO / Me-4PACz / PK	PECVD / PECVD / sputter / spin-coat / spin-coat	not at RJ: FBPAc at PK /C_{60} interface	31.25	1.91	79.8	20.47	1.2 / 1.1677	NREL	MPPT after 1 year inert storage, 80% after 66 h, air, 65 °C	10.1126/science.adg0091	Chin X.Y. et al., Science 381, 59–63 (2023)
8	Aydin 2023	a-Si / nc-Si / IZO / 2PACz / PK	PECVD / PECVD / RF sputter / spin-coat / spin-coat	ultrathin IZO at RJ	32.7 (32.5 stab.)	1.947	80.0	22.16	1.055	JET	90% after 870 h, MPPT, RT	10.1038/s41586-023-06667-4	Aydin E. et al., Nature 623, 732–738 (2023)
9	Ugur 2024	a-Si / nc-Si / IZO / Me-4PACz / PK	PECVD / PECVD / RF sputter / spin-coat / spin-coat	not at RJ: in-situ THTZ formation for PK	33.9 (33.7 stab.)	1.974	81.33	21.167	1.0035	ESTI	>90% after 1000 h, MPPT, 25 °C	10.1126/science.adp1621	Ugur E. et al., Science 385, 533–538 (2024)
10	Turkay 2024	a-Si / nc-Si / nc-SiO_x / ITO / Me-4PACz / SiO_2-NP / PK	PECVD / PECVD / PECVD / sputter / spin-coat / spin-coat / spin-coat	SiO_2-NP sublayer between ITO and SAM	30.93	1.953	79.9	19.812	1.17	Fraunhofer ISE	>85% after 84 h, no encapsulation, light cycle, ISOS-LC-1, ambient, 25 °C, 30-50% RH	10.1016/j.joule.2024.04.015	Turkay D. et al., Joule 8, 1735–1753 (2024)
11	Kan 2024	a-Si / nc-SiO_x / ITO / CuSCN / PK	PECVD / PECVD / sputter / co-spin-coat	inorganic CuSCN grains replaces SAM	31.38 (31.02 stab.)	1.927	78.59	20.666	0.9972	SIMIT	93.8% after 1200 h (45 °C); 90.2% after 1000 h (85°C/85% RH), both MPPT	10.1038/s41566-024-01561-5	Kan C. et al., Nat. Photon. 19, 63–70 (2025)
12	Liu 2024	a-Si / nc-SiO_x / IZO / MeO-4PACz / PK	PECVD / PECVD / sputter / spin-coat / spin-coat	not at RJ: bilayer passivation with LiF and EDAI; double-side textured SHJ cell	33.89	1.9657	83.0	20.761	1.004	NREL	80% after 1200 h, MPPT, RT	10.1038/s41586-024-07997-7	Liu J. et al., Nature 635, 596–603 (2024)
13	Liu 2024	a-Si / a-Si / ITO / MeO-2PACz / PK	PECVD / PECVD / sputter / spin-coat / evap.	not at RJ: evap. 3D/3D PK heterojunction	32.13 (31.5 stab.)	1.87	83.33	20.65	1.015	SIMIT	>95% after 800 h, ISOS-L-1, 30 °C, 35% RH; >94%	10.1016/j.joule.2024.06.	Liu Z. et al., Joule 8, 2834–2850 (2024)

#	1st auth or / Year	RJ stack (and adjacent layers, manufacture bottom to top)	RJ Deposition	RJ Key innovation	PCE (%)	V_{oc} (V)	FF (%)	J_{sc} (mA/cm²)	Active / Aperture area (cm²)	Cert. body	Stability metrics	DOI	Citation
					(lab if no certified report)								
											after 500 h ISOS-L-2, 65 °C, both MPPT	015	
14	Chen 2024	a-Si / a-Si / ITO / poly-TPD / PK	PECVD / PECVD / sputter / spin-coat / spin-coat	not at RJ: OA stabilization of WBG PK	32.2 (32.0 stab.)	1.942	81.27	19.56	0.95884	NIM	98.3% after 1301 h, ISOS-L-1; >90% after 800 h, ISOS-L2, both MPPT	10.1126 /science .ado910 4	Chen H., Yang C. et al., Science 385, 554–560 (2024)
15	Liu 2024	a-Si / nc-Si (n) / nc-Si (p) / Spiro-TTP / PK	PECVD /PECVD	nc-Si tunneling junction	30.05 (29.4 stab.)	1.81	82.91	20.01	0.5003	n.r.	>98% after 700 h, 25 °C, 30-40% RH, MPPT; >90% after 1500 h, 85°C in N_2, MPPT	10.1007 /s40820 -024- 01406-4	Liu J., Shi B., Xu Q. et al., Nano-Micro Lett. 16, 189 (2024)
16	Choz as-Barri entos 2025	a-Si / (C_{60} (n) / TaTm (p) / TaTm-CS9 / TaTm / PK	PECVD / Full thermal evap.	all-organic RJ with n-doped C60 (+PhIM) and p-doped TaTm (+F6-TCNNQ)	22.19	1.78	69.86	17.81	n.r.	n.r.	n.r.	10.1021 /acsene rgylett.5 c00155	Chozas-Barrientos S. et al., ACS Energy Lett. 10, 1733–1740 (2025)
17	Kore 2025	a-Si / ITO / 2PACz / TaTm / PK	PECVD / sputter / evap. / evap. / spin-coat	sputter ITO with evap. organic RJ (2PACz + TaTm)	29.6 (29.6 stab.)	1.848	79.6	20.1	1	n.r.	Only <60 min MPPT or storage	10.1039 /D4EE0 3899A	Kore B.P., Er-raji O., Fischer O. et al., EES 18, 354–366 (2025)
18	Er-raji 2025	a-Si / ITO / Me-4PACz / PK	PECVD / sputter / spin-coat / evap.-spin-coat	not at RJ: electron accumulation at C_{60} interface increases PCE	(31.6 stab.)	1.925	80.9	20.30	0.9961	Fraunhofer ISE	83.4% after 1000 h ISOS-D-3, 85°C, 85% RH, MPPT	10.1126 /science .adx174 5	Er-raji O. et al., Science 390, eadx1745 (2025)
19	Zhan g 2025	a-Si / nc-Si / IZO / CL-SAM / PK	PECVD / PECVD / sputter / spin-coat / spin-coat	cross-linked SAM	33.8 (33.61 stab.)	1.974	81.68	21.888	1	NPCC	>97% after 1200 h ISOS-L-2, 65°C, MPPT	10.1126 /science .ady687 4	Zhang X., Luo Y. et al., Science 390, 837–842 (2025)
20	Wan g 2025	a-Si / nc-SiO_x / RPD ICO:H / Me-4PACz / PK	PECVD / PECVD / RPD sputter / spin-coat / spin-coat	reactive-plasma-deposited Ce and H doped ICO	33.6 (33.2 stab.)	2.015	81.9	20.36	1	CPVT	>90% after 1000 h, 85°C, 85% RH, MPPT	10.1038 /s41586 -025- 09849-4	Wang S. et al., Nature 649, 59–64 (2026)
21	Liu 2025	a-Si / ITO / NiOx / Me-4PACz / np-Al_2O_3 / PK	PECVD / sputter / spin-coat / spin-coat / spray-coat / spin-coat	spray-coat Al_2O_3 on SAM enhances PK coverage	32.69	1.959	82	20.43	1.0037	NIMC	only >90% after 500 h, RT, N_2 atmosphere, MPPT	10.1038 /s41467 -025- 64546-0	Nat. Commun. (2025)
22	Zhan g 2025	a-Si / nc-SiO_x / ITO / SiOx nanospheres / 2PACz / PK	PECVD / PECVD / sputter / spin-coat / spin-coat / spin-coat	localized submicron contacts through SiO_x nanospheres	33.15 (33.08 stab.)	1.95	81.07	20.92	1	SIMIT	86.7% after 1100 h, ISOS-D3, 85°C, 85% RH; 91.7% after 1000 h ISOS-L-2I, 80 °C, N_2, MPPT	10.1038 /s41467 -025- 62389-3	Nat. Commun. (2025)
23	Longi 2025	n.r.	n.r.	n.r.	35	2.007	84.3	20.76	1.0028	NREL	n.r.	press release	LONGi press release + NREL chart (on April 2026)
			Perovskite							Perovskite			
24	Yu 2020	C_{60} / SnO_{2-x} / PK	evap. / low temp. ALD / spin-coat	simplified 2 layer RJ	24.6	2.03	79.7	15.2	0.059	n.r.	94% after 1000 h, ambient, RT. MPPT	10.1038 /s41560 -020- 0657-y	Yu Z. et al., Nat. Energy 5, 657–665 (2020)
25	Xiao 2020	C_{60} / SnO_2 / Au / PEDOT:PSS / PK	Evap. / ALD / evap. / spin-coat / spin-coat	not at RJ: improved Sn oxidation stability through zwitterion	24.2 (24.2 stab.)	1.986	76.63	16.58	1.041	JET	88% after 500 h, 54-60 °C, ambient, MPPT	10.1038 /s41560 -020- 00705-5	Xiao K. et al., Nat. Energy 5, 870-880 (2020)
26	Datta 2022	C_{60} / SnO_x / Au / PEDOT:PSS / PK	evap. / sALD / evap. / spin-coat	not at RJ: interfacial PK passivation	23.1 (23.0	1.95	75	15.8	0.09	n.r.	n.r.	10.1002 /adma.2	Datta K. et al., Adv. Mater. 34, 2110053

#	1st author / Year	RJ stack (and adjacent layers, manufacture bottom to top)	RJ Deposition	RJ Key innovation	PCE (%)	V_{oc} (V)	FF (%)	J_{sc} (mA/cm²)	Active / Aperture area (cm²)	Cert. body	Stability metrics	DOI	Citation
					(lab if no certified report)								
					stab.)							02110053	(2022)
27	Lin 2022	C_{60} / SnO_2 / Au / PEDOT:PSS / PK	evap. / ALD / evap. / spin-coat / spin-coat	not at RJ: PK passivation CF3-PA	26.3 (26.4 stab.)	2.044	77.8	16.53	0.04937	JET	90% after 600 h, ambient, RT, MPPT	10.1038/s41586-021-04372-8	Lin R. et al., Nature 603, 73-78 (2022)
28	Zhou 2022	PCBM / SnO_2 / PEDOT:PSS / PK	spin-coat / ALD / spin-coat / spin-coat	metal-free RJ	23.65	2.05	78.7	14.66	0.09	n.r.	only storage reported	10.1021/acsenergylett.2c02156	Zhou X. et al., ACS Energy Lett. 8, 502–512 (2023)
29	Zhu 2023	C_{60} / SnO_2 / Au / D-A SAM / PK	evap. / ALD / evap. / spin-coat / spin-coat	donor-acceptor SAM replacing PEDOT:PSS	26.3	2.105	82.3	15.21	0.0574	JET	80% after 301 h, ambient, RT, MPPT	10.1038/s41560-023-01274-z	Zhu J. et al., Nat. Energy 8, 714-724 (2023)
30	He 2023	C_{60} / SnO_2 / IZO / PEDOT:PSS / PK	evap. / ALD / sputter / spin-coat / spin-coat	not at RJ: 4PADCB for WBG improvement	26.6 (26.4 stab.)	2.119	82.4	15.91	1.044	JET	80% after 415 h, ambient, MPPT	10.1038/s41586-023-05992-y	He R. et al., Nature 618, 80–86 (2023)
31	Lin 2023	C_{60} / SnO_2 / Au / PEDOT:PSS / PK	evap. / ALD / evap. / spin-coat / spin-coat	not at RJ: bilayer PK heterojunction at NBG	28.0 (28.0 stab.)	2.125	80.3	16.42	0.049 / 0.04952	JET	93% after 600 h, ambient, MPPT	10.1038/s41586-023-06278-z	Lin R. et al., Nature 620, 994–1000 (2023)
32	Liu 2023	C_{60} / SnO_2 / ITO NCs / 2PACz / PK	evap. / ALD / spin-coat / spin-coat / spin-coat	SAM RJ without PEDOT:PSS	28.1	2.11	79.5	16.7	0.049	n.r.	90% after 500 h, ambient, 45 °C, MPPT	10.1002/anie.202313374	Liu C. et al., Angew. Chem. Int. Ed. 62, e202313374 (2023)
33	Pan 2024	C_{60} / SnO_2 / Au / PEDOT:PSS / PK	evap. / ALD / evap. / spin-coat / spin-coat	not at RJ: surface chemical polishing on Sn-Pb minimizes non-radiative loss	28.80	2.13	84.19	16.06	0.0871	n.r.	79.7% after 550 h, MPPT	10.1038/s41467-024-51703-0	Pan Y. et al., Nat. Commun. 15, 7335 (2024)
34	Wang 2024	C_{60} / SnO_2 / Au / PEDOT:PSS / PK	evap. / ALD / evap. / spin-coat / spin-coat	not at RJ: 2D PK layer homogenizes WBG/ETL interface	28.5	2.17	80.2	16.4	1.05	n.r.	n.r.	10.1038/s41586-024-08158-6	Wang Y. et al., Nature 635, 867–873 (2024)
35	Gao 2024	C_{60} / SnO_2 / Au / PEDOT:PSS / PK	evap. / ALD / evap. / blade-coat / blade-coat	not at RJ: notable Module size up-scale result	24.9 (24.5 stab.)	17.22	78.3	1.722	20.25	JET	80% after 330 h, 50°C, ambient, MPPT; 90% after 2000 h, 85% RH; 80% after 200 h, 85% °C, 85% RH; 86% after 200 thermal cycles -40°C to 85°C	10.1126/science.adj6088	Gao et al., Science 383, 6685, 855-859 (2024)
36	Liu 2025	C_{60} / SnO_x / Au / PEDOT:PSS / PK	evap. / ALD / evap. / spin-coat / spin-coat	not at RJ: 2D PK template for (100) WBG orientation	29.7	2.175	83.3	16.4	0.049 / 0.04888	JET	90% after 750 h, MPPT	10.1038/s41563-024-02073-x	Liu Z., Lin R., Wei M. et al., Nat. Mater. 24, 252–259 (2025)
37	Lian 2025	PCBM / SnO_2 / Au / PEDOT:PSS / PK	spin-coat / ALD / evap. / spin-coat	not at RJ: crown-ether passivation	28.44	2.14	81.31	16.36	0.1017 / 0.0678	SCM	only WBG MPPT reported	10.1038/s41467-025-62391-9	Lian X. et al., Nat. Commun. 16, 7173 (2025)
38	Fitzsimmons 2025	C_{60} / GO / 2PACz / PK	evap / spin-coat / spin-coat / spin-coat	graphene oxide with SAM replaces Au with PEDOT:PSS	23.3	1.94	77.7	15.4	0.12	n.r.	>100% after 100 h, 25 °C, N_2, MPPT	10.1021/acsenergylett.4c03065	Fitzsimmons M.R. et al., ACS Energy Lett. 10, 713–725 (2025)
39	Wei 2025	C_{60} / Cr / ITO / PEDOT:PSS / PK	evap. / evap. / sputter / spin-coat / spin-coat	thin Cr acts as sputter-damage barrier, no ALD or Au	26.56	2.075	80.6	15.88	0.09	n.r.	90% after 562 hours MPPT	10.1039/D5TA04259C	J. Mater. Chem. A (2025)

#	1st auth or / Year	RJ stack (and adjacent layers, manufacture bottom to top)	RJ Deposition	RJ Key innovation	PCE (%)	V_{oc} (V)	FF (%)	J_{sc} (mA/cm²)	Active / Aperture area (cm²)	Cert. body	Stability metrics	DOI	Citation
					(lab if no certified report)								
40	Lin 2025	C_{60} / SnO_x / Au / PEDOT:PSS / SA / PK	evap. / ALD / evap. / spin-coat / spin-coat / spin-coat	dipolar molecule tunes energy alignment for ohmic junction	30.6 / (30.1 stab.)	2.211	83.4	16.6	0.049 / 0.04934	JET	87% after 1,025 hours MPPT	10.1038/s41586-025-09773-7	Lin R., Gao H., Lou J., Tan H. et al., Nature 648, 600–606 (2025)
41	Sun 2026	C_{60} / SnO_2 / Au / P3CT-Cs / PK	evap. / ALD / evap. / spin-coat / spin-coat	not at RJ: CsOH forms stabilizing oxides on Sn–Pb against photothermal degradation	28.56	2.126	82.61	16.23	0.0731	SIMIT	90.3% after 500 h, ISOS-L-3 (65 °C); >80% after 150 h, ISOS-L-3 (85 °C) MPPT	10.1038/s41566-025-01815-w	Sun N., Fu S., Li Y. et al., Nat. Photon. 20, 273–279 (2026)
42	Wang 2026	C_{60} / SnO_2 / Au / PEDOT:PSS / PK	evap. / ALD / evap. / spin-coat / spin-coat	SAM with H-bond network replacing PEDOT:PSS	29.38 (28.4 stab.)	2.114	85.41	16.9	0.0686	SIMIT	90% after 638 h, N_2 ISOS-L-1, MPPT; 90% after 420 h, ambient ISOS-L-1, MPPT	10.1038/s41560-026-01964-4	Wang et al., Nat. Energy 11, 436-448 (2026)
		Silicon						Perovskite				Perovskite	
43	Choi 2023	RJ1: ITO /PTAA/ PK<br><br>RJ2: C_{60} / PEIE / ITO / SAM	RJ1: Sputter/ spin-coat<br><br>RJ2: spin-coat / Sputter/ spin-coat	ALD-free, not at RJ: less damaging PK solvents	22.23	2.78	78.6	10.18	n.r.	n.r.	n.r.	10.1021/acsenergylett.3c00919	ACS Energy Lett. 2023, 8, 7, 3141–3146
44	Liu 2024	RJ1: a-Si / nc-SiOx / ITO / Me-4PACz / PK<br><br>RJ2: C_{60} / SnO_2 / ITO / NiO_x / Me-4PACz / PK	RJ1: PECVD / PECVD / sputter / spin-coat / spin-coat<br>RJ2: evap. / ALD / sputter / spin-coat / spin-coat / spin-coat	not at RJ: OCN additive for 1.93 eV PK	27.10	3.145	78.08	11.58	1 / 0.9988	SIMIT	96% after 700 h, ISOS-D-3 (65 °C, 85% RH); 80% after 300 h, ISOS-L-3 (65°C, 50% RH)	10.1038/s41586-024-07226-1	Liu S. et al., Nature 628, 306–312 (2024)
45	Xu F. 2024	RJ1: a-Si / IZO / MeO-2PACz / PK<br><br>RJ2: C_{60} / SnO_2 / IZO / NiO_x / 2PACz	RJ1: Sputter/ spin-coat<br><br>RJ2: ALD/ Sputter/ spin-coat	not at RJ: KSCN additive	26.4 (26.2 stab.)	3.04	72.9	11.9	1	n.r.	98% after 300 h, 25 °C, in N_2, MPPT	10.1016/j.joule.2023.11.018	Xu F. et al., Joule 8, 224–240 (2024)
46	Li F. 2024	RJ1 : ITO / NiOx / 2PACz<br>RJ2 : LiF / C60 / ALD-SnOx / IZO	evap. / ALD / sputter / spin	Early certified PK/PK/Si 3J; Voc ~3.0 V, 1 cm² aperture<br>RbCl alloying in the 1.96 eV wide-bandgap top perovskite	25.0 / 24.19	2.995 / n.r.	71.0 / n.r.	11.76 / n.r.	1.04 / 1.04	NIMTT	unencapsulated tandem retained 86.8% of initial PCE after 100 h MPP under one-sun LED at 25 °C, 30 ± 10% RH	10.1002/adma.202311595	Li F. et al., Adv. Mater. 36, 2311595 (2024)
47	Heydarian 2025	RJ1: a-Si / ITO / PTAA / PFN / PK<br><br>RJ2: C_{60} / SnO_x / ZTO / 2PACz / PK	RJ1: PECVD / sputter / spin-coat / spin-coat / spin-coat<br><br>RJ2: evap. / ALD / sputter / spin-coat / spin-coat	indium-free sputtered RL for PK/PK/Si 3J	21.9	3.10	84.9	8.3	1	n.r.	n.r.	10.1002/smll.202511646	Heydarian M. et al., Small (2025) e202511646
48	Hu S. 2025	All RJs: C60 / ALD-SnOx (~20 nm) / sputter IZO (~10 nm) — universal RJ + PEDOT:PSS for Sn–Pb	Evap+ALD+sputter+spin	Amino-acid-salt (e.g., 4-FPhA·HCl) precursor chemistry enables Sn–Pb; universal ALD-SnOx/IZO RJ	27.28	3.428	78.4	10.2	1.003	AIST	T80 = 860 h (encapsulated, ~RH45)	10.1038/s41586-024-08546-y	Hu S., Wang J., Zhao P. et al., Nature 639, 93–101 (2025)
49	Artuk 2026	RJ1: a-Si / nc-Si / ITO / Me-4PACz:2PACz / SiO_x-np / PK<br><br>RJ2: C_{60} / SnO_x / IZO / Me-4PACz:4PABCz / SiO_x-np / PK	RJ1: PECVD / PECVD / sputter / spin-coat / spin-coat / spin-coat<br><br>RJ2: evap. / ALD / sputter / spin-coat / spin-coat	SiO_x nanoparticles improve current balance; not at RJ: perovskites optimized	30.44 (30.02 stab.)	3.207	81.31	11.373	0.9743	SIMIT	86.2% after 500 h, 85 °C, 85% RH, MPPT	10.1038/s41586-026-10385-y	Artuk K., et al., Nature 653, 90-97 (2026)

#	1st author / Year	RJ stack (and adjacent layers, manufacture bottom to top)	RJ Deposition	RJ Key innovation	PCE (%)	V_{oc} (V)	FF (%)	J_{sc} (mA/cm²)	Active / Aperture area (cm²)	Cert. body	Stability metrics	DOI	Citation
					(lab if no certified report)								
			Perovskite				Perovskite				Perovskite		Perovskite
50	Wang 2023	RJ1: PCBM / PEIE / SnO_x / ITO / NiO_x / PACz / PK<br><br>RJ2: PCBM / PEIE / SnO_x / Au / PEDOT:PSS	RJ1: spin-coat / spin-coat / ALD / sputter / spin-coat / spin-coat / spin-coat<br><br>RJ2: spin-coat / spin-coat / ALD / evap. / spin-coat	not at RJ: suppression of phase segregation	23.29	3.181	76.2	9.61	0.0505	NREL	80% after 420 h, RT, ambient, MPPT	10.1038/s41586-023-06006-7	Wang Z., et al., Nature 618, 74-79 (2023)
51	Wang 2024	RJ1: PCBM / PEI / SnO_x / ITO / NiO_x / Me-4PACz / PK<br><br>RJ2: C_{60} / SnO_x / Au / PEDOT:PSS / PK	RJ1: spin-coat / spin-coat / ALD / sputter / spin-coat / spin-coat / spin-coat<br><br>RJ2: evap. / ALD / evap. / spin-coat / spin-coat	not at RJ: halide homogenization in WBG sub-cell	23.87	3.267	80.3	9.0921	0.0508	NREL	80% after 200 h, ambient, RT, MPPT	10.1038/s41560-023-01406-5	Wang J., et al., Nat. Energy 9, 70-80 (2024)
52	Hu S. 2025	All RJs: C_{60} / SnO_x / IZO + PEDOT:PSS for Sn–Pb	evap. / ALD / sputter / spin-coat	not at RJ: First all-PK 4J; bandgap 2.26eV/1.80eV/1.55eV/1.26 eV	27.4 / 26.9	4.87 / 4.90	81 / 73	7.0 / 7.6	0.25 / 1	n.r.	n.r.	10.1038/s41586-024-08546-y	Hu S. et al., Nature 639, 93–101 (2025)
				CIGS							Perovskite		
53	Todorov 2015	PEDOT:PSS / ITO ~30 nm RJ / CdS / CIGS	Sputter ITO; CBD CdS	ZnO-free monolithic interconnection using ITO directly on CdS, combined with in situ vapor halide-exchange bandgap engineering	10.9 /	1.45 /	56.6 /	12.7 /	0.40	n.r.	n.r.	10.1002/aenm.201500799	Todorov T. et al., Adv. Energy Mater. 5, 1500799 (2015)
54	Han 2018	PTAA / polished ITO / BZO / i-ZnO / CdS	Sputter + CMP polishing+ spin	First ≥20% monolithic PK/CIGS; Nanoscale interface engineering of the CIGS surface via ITO + CMP smoothing, plus heavily doped PTAA HTL	22.43 / 22.4	1.774	73.1	17.3	0.042	NREL	Unencapsulated tandem retained 88% after 500 h under continuous 1-sun MPP tracking at 30 °C ambient; recovered to 93% after 12 h dark rest	10.1126/science.aat5055	Han Q. et al., Science 361, 904–908 (2018)
55	Al-Ashouri/Jost 2019	SAM (2PACz-type) directly on rough as-deposited CIGS — no ALD, no CMP	Solution SAM	Conformal SAM hole contact on rough as-deposited CIGS	23.16 ; 23.26 (stabilized)	1.68	71.9.	19.17	1.0347	Fraunhofer ISE	n.r.	10.1039/C9EE02268F	Al-Ashouri A., Magomedov A., Albrecht S. et al., EES 12, 3356–3369 (2019)
56	Jost 2019	ZnO:Al / ALD-NiOx / PTAA	ALD NiOx; spin PTAA	Conformal NiOx/PTAA bilayer HTL on rough as-grown CIGSe	21.6	1.58	76.0	18.0	0.778	n.r.	n.r.	10.1021/acsenergylett.9b00135	Jost M. et al., ACS Energy Lett. 4, 583–590 (2019)
57	Jost 2022	ZnO:Al / Me-4PACz	Sputter; solution SAM (dip coating)	SAM-based HTL integration on rough, non-planarized CIGS, combined with PEAI additive in perovskite	24.2	1.77	71.2	18.8	1.04	Fraunhofer ISE	n.r.	10.1021/acsenergylett.2c00274	Jost M. et al., ACS Energy Lett. 7, 1298–1307 (2022)
58	Kafedjiska 2023	i-ZO/AZO/NiO:Cu/SAM	i-ZO, AZO, NiO:Cu: sputter	Cu-doped NiO + SAM on CIGS	23.4	1.72	71	19.5	n.r.	n.r.	n.r.	10.1002/adfm.202302924	Kafedjiska I. et al., Adv. Funct. Mater. 33, 2302924 (2023)
59	Jost 2025	AZO / Au / NiO_x / 4PADCB	Sputter AZO; evap Au; ALD NiOx; spin SAM	Composite IRL (AZO/Au/NiOx/4PAD	30.71	1.745	80.2	21.76	0.15	n.r.	T93.9 = 663h (unencap ; N2 ;	10.1038/s41467	Jost M. et al., Nat. Commun. (2025)

#	1st auth or / Year	RJ stack (and adjacent layers, manufacture bottom to top)	RJ Deposition	RJ Key innovation	PCE (%)	V_{oc} (V)	FF (%)	J_{sc} (mA/cm²)	Active / Aperture area (cm²)	Cert. body	Stability metrics	DOI	Citation
					(lab if no certified report)								
				CB)							25oC)	-025-67350-y	
60	Lin 2025	ITO / NiO_x / mixed SAM (MeO-2PACz+Me-4PACz 85:15 v/v)	ITO sputter ; NiOx, SAM spin	Defect-passivation-failure-resistant TAR 3 passivator in the WBG perovskite,	28.23	1.922	77.19	19.03	0.21	SIMIT	MPPT : no degradation after 420 h in air.	10.1038/s41560-025-01761-5	Lin Y. et al., Nat. Energy 10, 824–835 (2025)
61	Pei 2025	ITO / NiO_x / SAM	ITO sputter ; NiOx, SAM spin	Interconnection stress-relief engineering	28.24	1.894	78.40	19.02	0.2018	SIMIT	T80 = 1123 h N2 ; ~40oC ;	10.1021/jacs.5c13264	Pei et al., JACS 147, 36815–36824 (2025)
62	Farias-Basulto 2025	i-ZnO/AZO/NiO_x/SAM	i-ZnO, AZO: sputter NiOx: spin-coated nanoparticle solution	Light management + bandgap engineering on 1.1 cm²	24.6 (cert. SS)	1.765	71.8	19.29	1.105	Fraunhofer ISE	maintained over 500 s (Stabilized power output, SPO)	10.1021/acsami.5c15458	Farias-Basulto G. et al., ACS AMI 17, 56250 (2025)
63	Ying 2025	ZnO:Al/i-ZnO/Me-4PACz	ZnO:Al/i-ZnO: sputter (purchased)	Flexible monolithic perovskite/CIGS, Antisolvent-seeded SAM	23.8 (cert. SS)	1.75	77.2	17.6	1.09	SIMIT	MPPT T90= 320 h, Nbc-93= 3000 (bending raius: 10 mm)	10.1038/s41560-025-01760-6	Ying Z. et al., Nat. Energy 10, 737 (2025)
64	Tang 2025	i-ZnO/AZO/NiO_x/4PADCB	i-ZnO, AZO, NiOx: sputter	Surface modification of RF sputtering-deposited NiOx films by SAM (4PADCB)	22.8	1.82	74.1	16.9	0.07	n.r.	MPPT T77= 400 h Nbc-95= 20000 (bending radius: 7 mm)	10.1002/aenm.202403682	Tang L. et al., Adv. Energy Mater. 15, 2403682 (2025)
			**Organic**								**Perovskite**		
65	Hiramoto 1990	Me-PTC/H2Pc/Au/Me-PTC/H2Pc (Au between organic p-n junction)	Au: thermal evaporation	First insertion of thin Au film between two unit cells	n.r.	0.78	n.r.	~0.018	n.r.	n.r.	n.r.	10.1246/cl.1990.327	Hiramoto M. et al., Chem. Lett. 19, 327 (1990)
66	Liu 2016	C_{60}-SB / Ag / MoO_3	C60-SB: spin-coated Ag, MoO3: thermal evaporation	Graded zwitterionic fullerene recombination layer for perovskite/polymer-BHJ	16.0	1.63	75.1	13.1	0.06	n.r.	n.r.	10.1021/acsami.5b12740	Liu Y. et al., ACS AMI 8, 7070 (2016)
67	Chen W. 2022	BCP / IZO / MoO_x	BCP, IZO, MoOx: sputter	First sputtered IZO with high NIR transmittance	22.95	2.065	74.7	14.88	0.08	SIMIT	MPPT T90> 500 h	10.1038/s41560-021-00966-8	Chen W. et al., Nat. Energy 7, 229 (2022)
68	Brinkmann 2022	SnO_x / InO_x/ MoO_x	SnOx, InOx: ALD MoOx: thermal evaporation	First ALD-metal oxide (InOx) ICL replacing Ag NPs	23.1	2.16	81.25	13.22	0.0174	Fraunhofer ISE	MPPT T80> 130 h	10.1038/s41586-022-04455-0	Brinkmann K.O. et al., Nature 604, 280 (2022)
69	Ma 2023	C_{60} / C-C1-P / ITO / MoO_3	evap. / spin-coat / sputter / evap.	C-C1-P to avoid ITO sputter damage	24.07	2.09	78.99	14.58	n.r.	n.r.	80% after 150 h, ambient, MPPT	10.1002/adma.202307502	Ma Z., et al., Adv. Mater., 35, 48, 2307502 (2023)
70	Maksudov 2024	SnO_2 / IZO / MoO_x / 2PACz	SnO2: ALD IZO: sputter MoOx: thermal evaporation SAM(2PACz): spin-coated	Replaces parasitic Ag NP RJ to IZO (near-zero NIR parasitic absorption)	23.61	2.10	77	14.56	n.r.	n.r.	maintained over 10 min of continuous illumination (MPPT)	10.1016/j.mser.2024.100802	Maksudov T. et al., Mater. Sci. Eng. R, 159, 100802 (2024)
71	Hwang 2024	i-ZnO / ITO(ion-milled)	i-ZnO, ITO: sputter	Intentionally deposited thicker ITO then flattened the top surface by ion-milling	17.5	1.46	68	17.67	n.r.	n.r.	maintained over 1000 s (Stabilized power output, SPO)	10.1002/eem2.12489	Hwang S. K. et al., Energy Environ. Mater. 7, e12489 (2024)
72	Jiang X.	SnO_x / Au	SnOx: ALD Au: thermal	Isomeric diammonium passivation (CyDAI2)	25.7	2.157	77.82	15.303	0.09	CNAS	MPPT T93= 700 h	10.1038/s41586	Jiang X. et al., Nature 635, 860

#	1st author / Year	RJ stack (and adjacent layers, manufacture bottom to top)	RJ Deposition	RJ Key innovation	PCE (%)	V_{oc} (V)	FF (%)	J_{sc} (mA/cm²)	Active / Aperture area (cm²)	Cert. body	Stability metrics	DOI	Citation
					(lab if no certified report)								
	2024		evaporation	at WBG perovskite surface								-024-08160-y	(2024)
73	An Y. 2025	SnO_2 / Au / Ph-4PACz / MoO_3 / Ph-4PACz	SnO2: ALD<br>Au, MoO3: thermal evaporation<br>SAM(Ph-4PACz): spin-coated	SAM sandwiched structure between Au and MoO3, Balances carrier transport by adopting SAM on MoO3 surface	24.53	2.216	80.65	13.725	0.04	Enli Tech.	MPPT T80> 650 h	10.1038/s41467-025-58047-3	An Y. et al., Nat. Commun. 16, 2759 (2025)
74	Wang Y.-D. 2025	SnO_x / ITO / MoO_x	SnOx: ALD<br>ITO: sputter<br>MoOx: thermal evaporation	Face-on stacking by using PACl additive in WBG perovskite for hybrid-deposited tandem	25.82	2.125	80.87	15.03	0.0503	SIMIT	MPPT T90= 400 h	10.1038/s41467-025-61404-x	Wang Y.-D. et al., Nat. Commun. 16, 6142 (2025)
75	Sun X. 2025	BCP / Ag / MoO_x / TCB-treated 4PADCB	BCP, Ag, MoOx: thermal evaporation<br>TCB-treated SAM(4PADCB): spin-coated	Highly ordered SAM by using opposite electrostatic potential TCB additive	26.09	2.131	81.90	14.95	n.r.	n.r.	maintained over 300 s (Stabilized power output, SPO)	10.1039/d4ee05533k	Sun X. et al., EES 18, 2536 (2025)
76	Jia Z. 2025	SnO_x / ITO / MoO_x	SnOx: ALD<br>ITO: sputter<br>MoOx: thermal evaporation	NIR-harvesting asymmetric non-fullerene acceptor P2EH-1V	26.4	2.124	82.78	15.02	1.019	SIMIT	MPPT T80= 783 h	10.1038/s41586-025-09181-x	Jia Z. et al., Nature 643, 104 (2025)
77	Tian 2025	PEDOT:F / Au / ZnO / PFN-Br	Au: thermal evaporation<br>PEDOT:F, ZnO NPs, PFN-Br: spin-coated	Surface-energy engineering tunes Au NP morphology to minimize LSPR loss	25.34	2.23	75.75	14.98	0.038	n.r.	maintained over 1000 s (Stabilized power output, SPO)	10.1038/s41467-024-55376-7	Tian J. et al., Nat. Commun. 16, 154 (2025)
78	He 2025	C_{60} / PEI / ITO / V_2O_5	evap. / spin-coat / sputter / evap.	V_2O_5 instead of MoO_3 improves optical and electric RJ properties	25.1	2.10	81.1	14.68	n.r.	n.r.	90% after 450 h, N_2, RT, MPPT	10.1038/s41467-025-57093-1	He Z., et al., Nat. Commun., 16, 1773 (2025)
		Organic					Perovskite				Perovskite		
79	Isikgor 2022	RJ1: C_{60} / BCP / IZO / NiO_X / N719<br><br>RJ2: C_{60} / BCP / IZO / MoO_3	RJ1: evap. / evap. / sputter / spin-coat / spin-coat<br><br>RJ2: evap. / evap. / sputter / evap.	First 3J perovskite-perovskite-organic	19.4	3.03	70.4	9.1	0.1	n.r.	n.r.	10.1021/acsenergylett.2c02340	Isikgor F. H., et al., ACS Energy Lett., 7, 12, 4469-4471 (2022)
